# 1899. The magnitude and durability of the antibody response to mRNA-based vaccination among SARS-CoV-2 seronegative and seropositive healthcare personnel

**DOI:** 10.1093/ofid/ofac492.1526

**Published:** 2022-12-15

**Authors:** Emily Ciccone, Deanna Zhu, Samuel Hawke, Rawan Ajeen, Annika Gunderson, Evans Lodge, Bonnie E Shook-Sa, Haley Abernathy, Haley Garrett, Elise King, Alena Markmann, Lakshmanane Premkumar, Jonathan J Juliano, Ross M Boyce, Allison Aiello

**Affiliations:** Division of Infectious Diseases, School of Medicine, University of North Carolina, Chapel Hill, North Carolina; Department of Epidemiology, Gillings School of Global Public Health, University of North Carolina; Department of Biostatistics, Gillings School of Global Public Health, University of North Carolina, Chapel Hill, North Carolina; Department of Epidemiology, Gillings School of Global Public Health, University of North Carolina; Department of Epidemiology, Gillings School of Global Public Health, University of North Carolina; Department of Epidemiology, Gillings School of Global Public Health, University of North Carolina; Department of Biostatistics, Gillings School of Global Public Health, University of North Carolina at Chapel Hill, Chapel Hill, North Carolina; Institute for Global Health and Infectious Diseases, University of North Carolina, Chapel Hill, North Carolina; Department of Epidemiology, Gillings School of Global Public Health, University of North Carolina; Institute for Global Health and Infectious Diseases, University of North Carolina, Chapel Hill, North Carolina; Division of Infectious Diseases, School of Medicine, University of North Carolina, Chapel Hill, North Carolina; Institute for Global Health and Infectious Diseases, University of North Carolina, Chapel Hill, North Carolina; University of North Carolina School of Medicine, Durham, NC; University of North Carolina at Chapel Hill, Chapel Hill, North Carolina; Department of Epidemiology, Gillings School of Global Public Health, University of North Carolina

## Abstract

**Background:**

Development of robust vaccination guidelines against SARS-CoV-2 requires an understanding of the longitudinal antibody (Ab) response to vaccination and interactions with natural infection. Here, we leveraged an observational cohort study of healthcare personnel (HCP) to study the impact of prior SARS-CoV-2 infection on Ab binding and neutralization after mRNA-based vaccination over a 13 month period.

**Methods:**

From July 2020 to February 2022, HCP at an academic medical center provided blood samples biweekly for 12 weeks and monthly thereafter. First and second vaccine doses became available in mid-December 2021 and boosters were available starting in October 2021. Individuals were excluded if they did not provide any samples, if baseline serostatus was unknown, and if they received a monoclonal Ab treatment for COVID. ELISA measured total immunoglobulin (Ig) and IgG binding to SARS-CoV-2 RBD. Neutralization was measured by live virus Nanoluc SARS-CoV-2ic assay.

Demographics, serostatus, and vaccinations for the total study population and the sub-sample of participants with pre- and post-vaccination antibody measurements.

**Figure:**
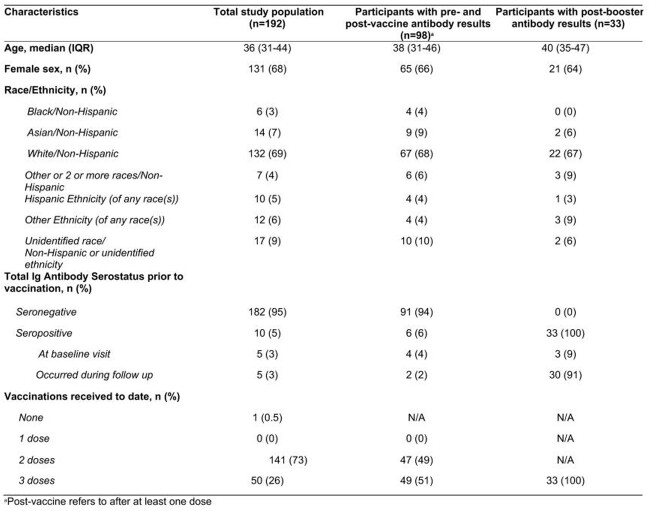

**Results:**

Of 213 participants, 192 met inclusion criteria. A majority had detectable IgG levels 8 months after a second dose. Prior to vaccination, median total Ig was higher among seropositive vs. seronegative participants (3.7 vs 1.0, p< 0.001). After a first dose, the median total Ig response was two-fold higher in seropositive compared to seronegative participants (13.8 vs. 7.0, p=0.009). A similar pattern was noted with IgG binding and neutralization. After the second dose, median IgG increased to similar levels in both seropositive and seronegative participants (22.1 vs. 21.2, p=0.8). Neutralization after the second dose was slightly higher in seropositive vs. seronegative participants (log10 3.1 vs. 2.5, p=0.075).

Durability of IgG responses after second dose of mRNA-based vaccination against SARS-CoV-2

**Figure:**
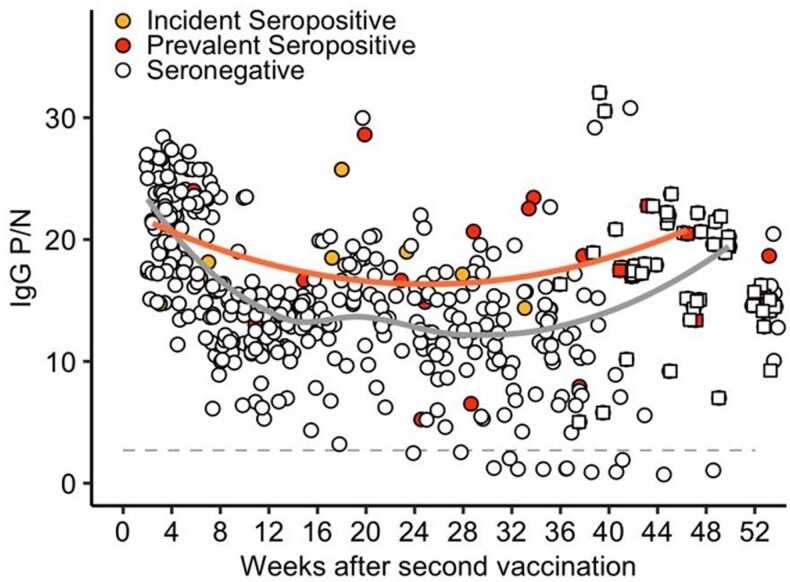

IgG P/N measurements after 5 days post-V2 for the entire study cohort (incident seropositive: yellow circles, prevalent seropositive (red circles), seronegative (open circles) are shown. The solid lines represent Loess curves for incident and prevalent seropositive participants combined (orange line) and those who were seronegative (grey line).

SARS-CoV-2-specific total Ig and IgG subtype responses among healthcare personnel before and after vaccination against SARS-CoV-2 with an mRNA-based vaccine.

**Figure:**
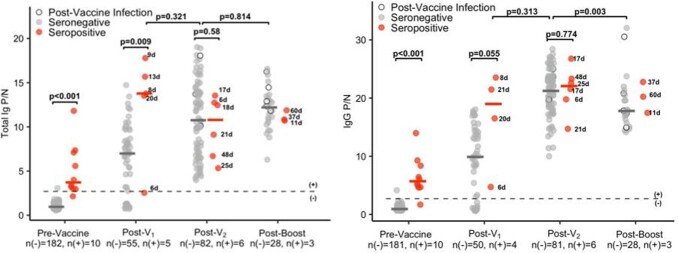

Total Ig P/N ratios at pre-vaccine, post-V1, post-vaccine 2 (post-V2), and post-booster dose (post-boost) timepoints by serostatus (seronegative: n(-), seropositive: n(+)) are shown in the left panel. For the pre-vaccine time point, the most recent antibody level prior to vaccination (for those who were vaccinated) or most recent antibody level overall (for those who were not vaccinated) is shown. For the post-vaccine time points, the first measurement after 5 days post-vaccination is included. Individuals who were infected with SARS-CoV-2 at any time after the first vaccine dose are shown as open circles with black outlines. The black numbers next to the circles indicate the number of days between vaccination and sample collection for seropositive individuals. SARS-CoV-2 specific IgG P/N ratios respectively at pre-vaccine, post-V1, post-V2, and post-boost timepoints by serostatus (seronegative: n(-), seropositive: n(+)) are shown in the right panel. One individual tested positive for SARS-CoV-2 by PCR shortly after the second vaccine dose (V2); post-V2 results were excluded for this participant. For the pre-vaccine time point, the most recent antibody level prior to vaccination (for those who were vaccinated) or most recent antibody level overall (for those who were not vaccinated) is shown. For the post-vaccine time points, the first measurement after 5 days post-vaccination is included. The dotted line is a P/N ratio of 2.4, the cut-off associated with 99.3% specificity (SARS-CoV-2 IgG-positive above the line, IgG-negative below). Individuals who were infected with SARS-CoV-2 at any time after the first vaccine dose are shown as open circles with black outlines. The black numbers next to the circles indicate the number of days between vaccination and sample collection for seropositive individuals.

SARS-CoV-2 D614G live virus neutralization among healthcare personnel by serostatus prior to vaccination.

**Figure:**
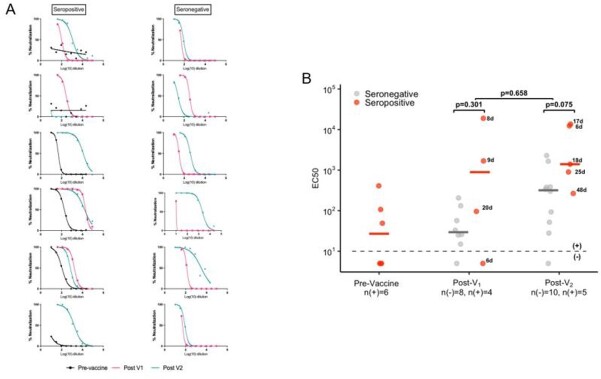

Example neutralization curves are shown in Panel A.

Panel B shows the SARS-CoV-2 D614G live virus neutralization titers displayed as EC50 for seropositive (prevalent and incident) individuals and a subset of seronegative individuals. Samples for seronegative individuals were selected by matching on age and time between vaccination and sample collection to the samples from seropositive individuals.

**Conclusion:**

Antibody responses after SARS-CoV-2 vaccination persist up to 1 year with wide individual variability. Though prior infection was associated with greater Ab responses after a first dose, it did not significantly modify responses after second and third doses. Still, we observed overall slightly higher Ab levels among individuals that had a prior infection before any one of the 3 doses of vaccine. These results suggest that immunity against SARS-CoV-2 prior to vaccination has a role in initial response but does not significantly modify circulating Ab titers after multiple doses of vaccination.

**Disclosures:**

**All Authors**: No reported disclosures.

